# The basic science of peri-implant bone healing

**DOI:** 10.4103/0019-5413.77129

**Published:** 2011

**Authors:** Paul RT Kuzyk, Emil H Schemitsch

**Affiliations:** 1 Division of Orthopedic Surgery, Department of Surgery, University of Toronto; 2 Division of Orthopedic Surgery, St. Michael’s Hospital, Toronto, ON, Canada

**Keywords:** Bone bonding, cementless implants, peri-implant bone healing

## Abstract

Given the popularity of cementless orthopedic implants, it is imperative for orthopedic surgeons to have a basic understanding of the process of peri-implant bone healing. Contact and distance osteogenesis have been used to explain peri-implant bone healing. In contact osteogenesis, *de novo* bone forms on the implant surface, while in distance osteogenesis, the bone grows from the old bone surface toward the implant surface in an appositional manner. Contact osteogenesis may lead to bone bonding if the surface of the implant displays the appropriate surface topography. The early stage of peri-implant bone healing is very important and involves the body’s initial response to a foreign material: protein adsorption, platelet activation, coagulation, and inflammation. This results in the formation of a stable fibrin clot that is a depot for growth factors and allows for osteoconduction. Osteoconduction is the migration and differentiation of osteogenic cells, such as pericytes, into osteoblasts. Osteoconduction allows for contact osteogenesis to occur at the implant surface. The late stage of healing involves the remodeling of this woven bone. In many respects, this process is similar to the bone healing occurring at a fracture site.

## INTRODUCTION

The success of total joint arthroplasty is dependant on the interface between the metal and bone. Sir John Charnley chose bone cement (polymethylmethacrylate) to create this interface.^1^ Although bone cement is still frequently used today for femoral stems, the cementless femoral stem has become the standard for primary total hip arthroplasty in young, active patients. Unlike cemented implants that rely on a cement mantle to fix metal to bone, cementless implants rely on bone healing to secure the implant to the host bone. Osseointegration has been used to describe the successful healing of an implant within a host bone.^2^ However, osseointegration is a confusing term because it does not have an exact definition in literature. Perhaps a better term to describe the ultimate goal of a cementless implant is bone bonding. Bone bonding is the ability of the bone to bond to the surface of a synthetic material, such as a titanium implant.^3^

The biological process behind bone bonding occurs within the normal human bone and the bones of all vertebrates, as a part of physiological bone remodeling. In bone remodeling, osteoclasts remove the old bone and in so doing provide a surface on the old bone with the appropriate topography, with which the newly formed bone may bond. This bonding occurs as the cement line interdigitates with the surface of the old bone. The cement line is the first matrix developed during *de novo* bone formation. Evidence suggests that this process may occur at an implant surface, and thus result in bone bonding, if the implant’s surface topography is three dimensionally complex, with pores and undercuts.^2^

Peri-implant bone healing is not limited to bone bonding and certainly stable implant fixation occurs in the absence of bone bonding, although bone bonding may be regarded as the ideal situation. This review will explore peri-implant bone healing in its early and late phases. The early phase of healing proceeds from hematoma formation to woven bone formation. The late phase of healing results in bone remodeling. This process is analogous to intramembranous healing at a fracture site.

## EARLY PERI-IMPLANT BONE HEALING

Peri-implant healing begins when the surgeon prepares the bone to accept the metal implant (e.g., reaming and rasping of the femoral canal for a femoral stem). Preparation of the bone in such a manner is important for implant healing because: (1) it allows for initial implant stability, and (2) it causes bleeding that leads to formation of a hematoma.

### Initial implant stability

Primary implant stability is a requirement for successful peri-implant healing.^4^ A stable implant has limited micromotion between the bone and implant, thereby allowing successful tissue growth around the implant (e.g., angiogenesis and osteogenesis).^5^ This is similar to a healing fracture. In the case of a fractured bone, instability between the bone ends, drives the cartilage formation over the bone formation.^6^,^7^ At the extreme, this leads to the development of pseudoarthrosis between the two bone ends. Le **et al**., shows that motion at a fracture site leads to prolonged expression of the Indian hedgehog gene, an important regulator of chondrocyte maturation.^8^ In the case of an implant, excessive motion has been shown to favor fibrous connective tissue ingrowth over bone ingrowth.^3^ Pilliar**et al**., suggest that micromotion greater than 150 *µ*m would lead to the attachment of fibrous tissue to the implant surface instead of bone.^9^ As a result, the authors suggest that early weight bearing on cementless orthopedic implants be restricted. Yet, in both healing fractures and implants, some degree of micromotion (probably less than 25 – 50 *µ*m) is required to promote bone formation.^10^

**Figure 1:**
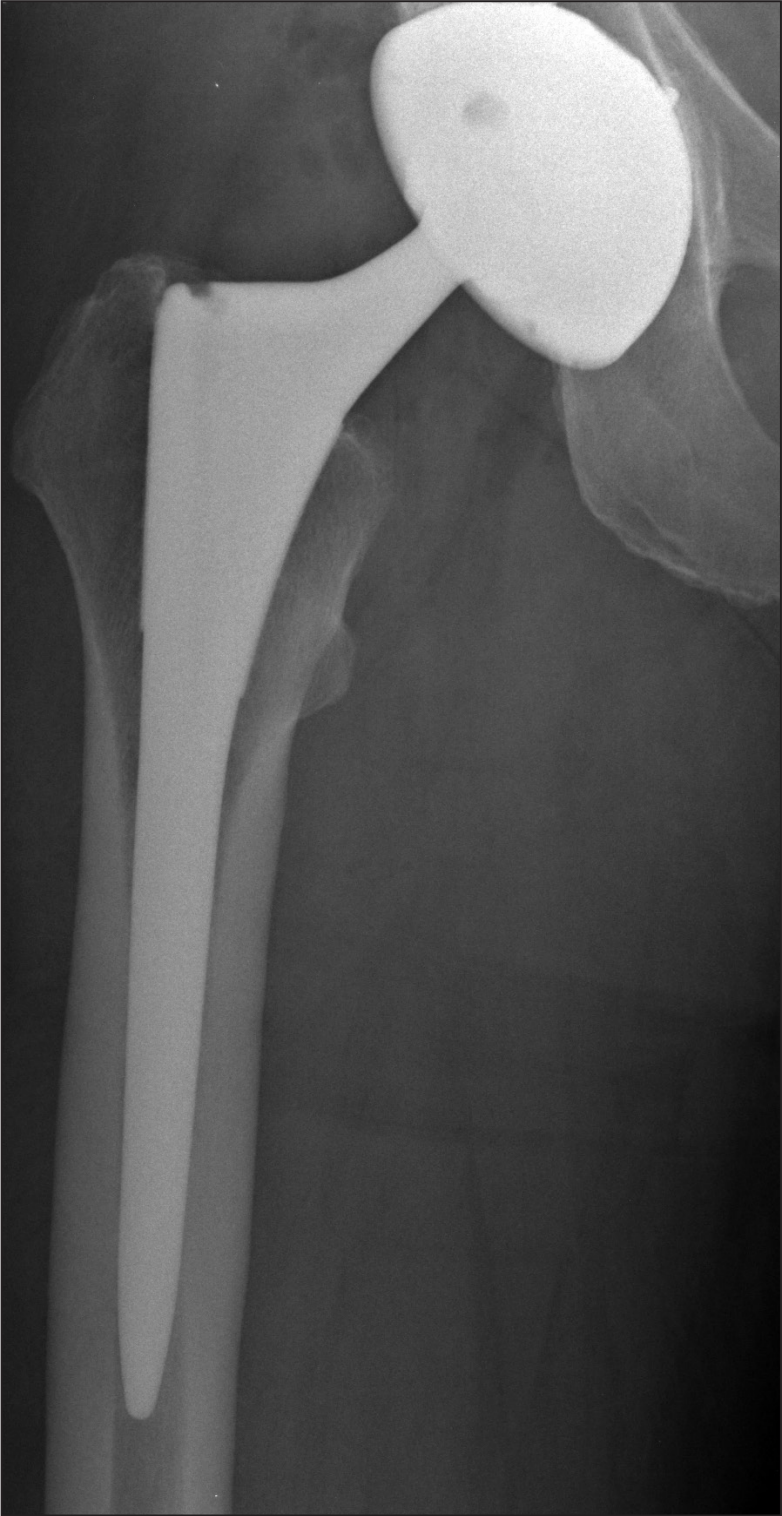
Radiograph of a well-fixed, tapered, cementless femoral stem at an eight-year follow-up

Primary implant stability is accomplished through a friction fit between the implant and the bone. A good example of this friction fit is a press-fit acetabular cup. There is a trade-off between stability and contact between the bone and the implant.^5^ Poor bone formation is observed when the implant is in close contact with the cortical bone.^11^ On the other hand, gaps greater than 500 *µ*m have been shown to reduce the quality and rate of bone formation.^5^,^11^ Recognition of the importance of primary implant stability has led to the development of anatomic and tapered cementless femoral stem designs. These stems mirror the shape of the proximal femur, allowing for a snug fit, without formation of gaps [Figure 1]. Such implants have had an excellent clinical track record, with a failure rate of less than 1% at the 10 and 15-year clinical follow-up.^12^–^14^

The surgical intervention required to introduce the implant and produce primary implant stability leads to hemorrhage from the bone and surrounding soft tissues. This leads to blood contact with the implant and hematoma formation.

### Blood contact with the implant

Blood is invariably the first tissue that the implant will contact when introduced into the bone. This contact results in a series of biological processes: protein deposition, coagulation, inflammation, and tissue formation. On the part of the host, these processes represent an evolutionary response to injury on introduction of a foreign material(i.e., the implant). Implant surface chemistry and topography influence these processes.^15^ We will begin by examining protein deposition on the implant.

A monolayer of protein is present on the implant surface only seconds after it contacts blood. It is this monolayer with which the platelets and mesenchymal cells will interact. Blood contains over 200 different proteins and only certain proteins from this milieu will appear on the implant surface to any appreciable extent.^16^ The composition of the protein monolayer is largely determined by the surface properties of the implant. Furthermore, the implant surface will determine the conformation of the proteins that become adsorbed. Proteins are charged molecules that change conformation (i.e., the protein’s three-dimensional shape) depending on their electrochemical environment. Therefore, surface charge characteristics of the implant are thought to determine a protein’s conformation. The conformation of the protein is important, as it determines whether certain bioactive peptide sequences located within the protein will be available for the incoming cells.^16^ An example of a bioactive peptide sequence is the arginine-glycine-aspartic acid (RGD) amino acid sequence. This sequence is responsible for mediating cell binding and integrin-mediated signaling for many different types of cells (e.g., endothelial cells, osteoclasts, and osteoblasts).

The types of proteins adsorbed onto the implant surface may determine the host’s response to the material and therefore may determine if successful peri-implant healing will occur. Fibronectin and vitronectin contain RGD sequences, and may therefore, interact with mesenchymal cells through their cell surface integrins.^17^ Fibrinogen, von Willebrand factor, complement, and IgG are also adsorbed onto the implant surface and are important for platelet activation, coagulation, and inflammation.

### Coagulation and platelet activation

Platelets are the first cells to contact the implant surface and have been shown on the implant surface within five seconds after contact with blood.^18^ Platelets are small cells formed from megakaryocytes and are activated through contact with foreign material (implant surface), injured endothelium, subendothelium or factors released by other platelets or cells (e.g., ADP, thromboxane A_2,_ and thrombin). The activation of platelets results in a number of important intracellular processes. Bioactive molecules stored in granules [e.g., ADP, platelet-derived growth factor (PDGF), histamines, and serotonin] within the platelet are released into the surrounding environment. P-selectin is expressed on the platelet’s membrane surface. P-selectin is a cell-surface glycoprotein that aids in platelet adhesion to neutrophils, monocytes, and leukocytes. Along with P-selectin expression, activated platelets form platelet microparticles. These microparticles bind to fibrinogen and fibrin, and are pro-coagulants. Platelet activation causes a significant change in the shape of the cell. This is important for coagulation as it allows for the expression of factors tenase and prothrombinase within the cell membrane. The change in shape also leads to the expression of adhesion receptors.^19^

Platelets contain membrane-bound adhesion receptors [i.e., glycoproteins (GPs) Ib and IIb/IIIa] on their surface. Platelet adhesion to an implant surface is mediated by these two receptors. The GP Ib receptor requires the von Willebrand factor as a coreceptor. It will only bind to the von Willebrand factor if the factor is immobilized (such as on the surface of an implant). The GP IIb/IIIa receptor binds to the adsorbed proteins on the implant surface (e.g., fibronectin, vitronectin, von Willebrand factor, and fibrinogen). The platelet must be activated to allow binding through the GP IIb/IIIa receptor to occur. Activation of the platelet causes conformational changes in the receptor that allow it to bind with the adsorbed proteins on the implant.^20^

Activation of multiple platelets causes them to aggregate and form a clot. Fibrinogen is an important plasma protein in this process and supports aggregation of platelets through Ca^2+^-dependant binding with the activated GP IIb/IIIa platelet receptors. Activated platelets catalyze the production of thrombin from prothrombin. (This occurs concurrently with the coagulation pathway). Thrombin then acts to stabilize the growing platelet thrombus through the production of a stable fibrin polymer from fribrinogen. This is accomplished with the addition of activated factor XIII to the fibrin polymer.^20^

Formation of a stable clot provides both the mechanical and biochemical components required for osteoconduction. Osteoconduction has been defined by Davies and Hosseini as the recruitment and migration of osteogenic cells.^21^ The fibrin clot is a three-dimensional provisional matrix with incorporated adhesive plasma proteins (e.g., fibronectin). This allows for cell adhesion and migration from the capillary bed toward the implant. Many signaling molecules are found within this clot: cytokines, chemoattractants, mitogens, and growth factors.^22^ The clot acts as a biodegradable depot for these chemicals — much like a tissue engineering scaffold loaded with growth factors. Platelets are an important source for the signaling molecules found within the fibrin clot (e.g., chemoattractants for neutrophils and monocytes).^23^,^24^ Tissue growth factors beta 1 and 2 are important signaling molecules found within platelets. Tissue growth factor beta 1 has recently been shown to induce the migration of osteoprogenitor cells through the SMAD signaling pathway.^25^

Platelets are activated by contacting the surface of the implant, and the degree of activation has been shown to be influenced by the implant topography. Kikuchi **et al**., have shown *in vitro* that implants with microtopographical features (i.e., an implant surface exhibiting features of≤3 *µ*m) displayed greater activation than surfaces that were smoother at this micron level.^26^ Furthermore, activation of platelets was influenced much more strongly by the presence of microtopographical features on the implant surface than by the presence of calcium phosphate.

Activation of platelets at the surface of the implant leads to a natural gradient for the signaling molecules (i.e., high concentration near the implant surface and low concentration near the cut edges of the host bone). Chemoattractants released from platelets may therefore influence migration of monocytes, neutrophils, and mesenchymal cells toward the implant surface.^27^ Thus, the fibrin clot is essential for mediating both inflammation and osteoconduction.^28^

### Inflammatory response and signaling molecules

The inflammatory response occurs concurrently and interacts with coagulation and platelet activation.^19^ Neutrophils and monocytes are important leukocytes in the inflammatory response. After platelets, these are the next cells to migrate into the peri-implant space. Neutrophils are the first to arrive, with peak levels at 24 to 48 hours. However, monocytes rapidly transform into macrophages, which are the dominant leukocytes after 48 hours.^22^ Leukocytes traveling within the capillaries surrounding the implant become activated in response to the cytokines released by the platelets (e.g., β-thromboglobulin and PDGF).^29^ Another important result of leukocyte activation is the release of inflammatory mediators. These mediators include cytokines such as IL-1, IL-6, IL-8, tumor necrosis factor α (TNF-α), and the macrophage colony stimulating factor.^22^

The hematoma of the peri-implant space is similar to a fracture hematoma, with expression of the same signaling molecules. The relative mRNA expression levels for the various signaling molecules of the fracture hematoma have been studied and show variations over time and with different stages of fracture healing.^7^ Inflammatory cytokines are the first signaling molecules to be expressed and seem to be required to initiate bone formation. Beyond recruitment of leukocytes, the role of these inflammatory cytokines in bone formation remains uncertain. Gerstenfeld **et al**., have shown that TNF-α null animals exhibit delayed intramembranous ossification within a fracture site, suggesting that TNF-α is required for proper mesenchymal cell recruitment and / or differentiation into osteogenic cells.^30^

Similar to mediators of inflammation, members of the tissue growth factor β (TGF-β) superfamily are also expressed within 24 hours of injury.^31^ Members of this family include bone morphogenic proteins (BMPs) and growth and differentiation factors (GDFs). These factors have been shown to promote bone formation at a fracture site. BMP-2 and BMP-7 are currently used clinically in North America, to enhance the healing of fracture nonunions.^32^ Osteoinductive factors (i.e., factors that drive the differentiation of osteogenic cells from mesenchymal cells) have also shown improved bone formation during peri-implant healing with *in vivo* animal models.^33^–^36^

Metabolically active osteogenic cells require a blood supply and therefore angiogenesis is essential. Angiogenic factors are expressed concomitantly with metallomatrix proteinase. Metallomatrix proteinase degrades the extracellular matrix surrounding the pre-existing capillaries, allowing for the sprouting of new vessels.^37^ Degradation of the extracellular matrix results in the release of angiogenic factors stored within the matrix. The vascular endothelial growth factor (VEGF) is an important mitogen for endothelial cells, which is stored within the extracellular matrix.^38^ VEGF stimulates endothelial cells within the pre-existing capillaries, to loosen their gap junctions, undergo cell division, and migrate to form new vessels. VEGF also mediates the differentiation of perivascular cells or pericytes, into endothelial cells and smooth muscle cells, for the development of new vessels. Futhermore, VEGF receptors have been found on osteoblasts, suggesting that this factor may also modulate osteoblast function.^39^ PDGF, angiopoietin, and the basic fibroblast growth factor (bFGF) have also been shown to be important factors for angiogenesis.^37^

The complex interaction of the signaling molecules within the peri-implant space results in recruitment, migration, and differentiation of mesenchymal cells (i.e., both osteoinduction and osteoconduction). These osteogenic cells will participate in the formation of a woven bone.

### Woven bone formation

Mesenchymal cells are recruited from the marrow, pericytes, and the cambium layer of the periosteum.^30^ Pericytes are considered as tissue-resident mesenchymal cells and are brought into the wound site concomitant with the process of angiogenesis.^40^ The mesenchymal cells migrate through the preliminary matrix of the fibrin clot toward the implant surface. This is probably mediated by the numerous factors released by platelets and leukocytes.^27^ As the mesenchymal cells move to the implant surface, these same factors cause the cells to differentiate into the osteoblastic lineage. These osteoprogenitor cells colonize the implant surface and begin secreting the matrix. This has been shown to occur within 24 hours after implantation, in a porcine model.^41^ The initial matrix secreted by these cells does not contain collagen.^15^

The matrix secreted by the osteoprogenitor cells that arrive at the implant surface forms the afibrillar interfacial zone. The thickness of this zone has been reported to vary from 0.2 to 0.5 *µ*m.^15^ This afibrillar interfacial zone, first described by Davies **et al**., is analogous to the cement line that outlines the osteons of the lamellar bone.^42^,^43^ It is electron-dense and consists of noncollagenous proteins (specifically osteopontin and bone sialoprotein) and proteoglycans from the plasma (osteonectin and α _2_HS-glycoprotein).^15^,^44^ The osteopontin and bone sialoprotein have nucleation sites for calcium phosphate mineralization. Thus, the afibrillar interfacial zone forms a noncollagenous, calcified layer on the surface of the implant. Beyond this layer, there is a collagenous compartment that consists predominantly of type I collagen. Mineralization of this collagenous compartment proceeds after mineralization of the afibrillar interfacial zone.^15^ Formation of the collagen compartment is accomplished by fully differentiated osteoblasts. The osteoblasts move backward, away from the advancing mineralization front; however, they are sometimes unable to escape and become enveloped.^3^ When this occurs, the osteoblast becomes an osteocyte within a bone lacuna. This process results in the formation of immature woven bone, proceeding in an appositional fashion from the implant’s surface to the cut edges of the bone. This process is known as ‘contact osteogenesis’.^28^

Formation of the bone may also occur in the opposite direction — proceeding from the cut bone surface toward the implant. This is termed as ‘distance osteogenesis’.^28^ In distance osteogenesis, the osteocytes within the bone edges cut during implantation, will die due to thermal necrosis. This extends to a depth of 100 to 500 *µ*m and the resultant dead bone is reabsorbed by the osteoclasts.^15^,^45^ Differentiating osteoblasts migrate to the surface of the reabsorbed bone and form a noncollagenous cement line similar to that on the implant surface.^3^ This is subsequently followed by the formation of a collagen containing layer by fully differentiated osteoblasts. Mineralization occurs from the cement line and into the collagen layer. This produces woven bone by apposition that extends from the cut bone surface and encroaches on the implant surface. Thus bone formation occurs in two opposite directions. Fluorochrome labeling of the bone suggests that contact osteogenesis occurs at a rate that is 30% faster than distance osteogenesis.^15^,^21^

Contact and distance osteogenesis result in immature woven bone formation around the implant. This provides secondary stabilization of the implant within the host bone. Thus, there is a changeover from the primary stabilization that results from a friction fit of the implant with the host bone at the time of implantation, to secondary stabilization that results from the formation of the woven bone around the implant.^46^ The primary stabilization declines over time, as the host bone that is in direct contact with the implant dies and is reabsorbed by osteoclasts. Secondary stability of the implant may result from bone bonding, if the surface topography of the implant allows for contact osteogenesis. If the implant’s surface topography is three-dimensionally complex, with pores and undercuts, the cement line may interdigitate with the implant surface and bone bonding occurs. If the implant surface does not have the appropriate topography, the bone simply grows to the implant via distance osteogenesis and bone bonding is not achieved.^47^

### Implant surface design and osseointegration

Success of a cementless metal implant is dependent on the speed of early peri-implant healing and the resultant mechanical strength of the bone-implant interface. Both these parameters may be enhanced through implant surface modifications. The two most commonly employed methods for improving the bone interfacing surface of implants include: (1) sintering of metallic beads or fibers over the implant surface, and (2) plasma spray deposition of metallic or ceramics onto the implant surface.

Sintered porous coating involves heating either Co-Cr-Mo or titanium alloy implants to high temperatures for sustained periods of time. Unfortunately, this may alter the mechanical properties of the implant. Heating tends to increase metal grain size within the implant (Mechanical strength of an implant is inversely proportional to the metal grain size). The result of sintering is a single- or multi- layered porous coating. Stabilization of the implant within the host bone is achieved through bone growth into the pores of the coating or bone ingrowth. For optimal bone ingrowth, the pores must be greater than 100 *µ*m in size.^48^

The plasma spray involves superheating either titanium or ceramic powder and spraying the resultant plasma onto the surface of a titanium alloy implant. The plasma hits the surface of the implant and rapidly solidifies, forming a deposit on the surface. This results in an irregular surface topography that may allow for bone bonding onto the implant surface. There is evidence to suggest that the implant surface becomes rougher and more complex after implantation. Edwards **et al**., have shown that smooth hydroxyapatite-coated implants developed a breakdown at the material grain boundaries and a rough surface after implantation in rabbit tibia.^49^

The plasma spray process is commonly used to coat implants with hydroxyapatite (HA). However, the superheating of HA during the plasma spray process results in a loss of the normal stoichiometry of HA. The resultant deposition on the implant surface is actually a mixture of different calcium phosphate phases (e.g., HA, tricalcium phosphate, tetra calcium phosphate, and amorphous calcium phosphate).^48^ Thus, HA plasma-sprayed implants should not be referred to as HA-coated implants, but rather calcium phosphate-coated implants. Both titanium- and HA plasma-sprayed coatings rely on bone bonding for implant fixation within the host bone, thus the strength of the final bony fixation is essentially the same for both coatings. However, the HA plasma-sprayed implants appear to have faster peri-implant healing than the titanium-sprayed implants. This enhanced osteoconductivity may be due to chemical factors(e.g., increase in peri-implant concentrations of Ca^2+^and PO_4_^3-^), but this is more likely due to the enhanced topography features of the calcium phosphate coating (e.g., improved platelet activation).^26^

## LATE REMODELING OF THE BONE-IMPLANT INTERFACE

Although remodeling of the bone is considered here as the late stage of peri-implant healing, remodeling occurs throughout the healing process and continuously in all bones of the body. Remodeling occurs in a defined sequence of events — activation of osteoclast cutting cones, removal of bone by osteoclasts, angiogenesis bringing pericytes, differentiation of pericytes into osteoblasts, and formation of bone by these osteoblasts.^50^ This has been referred to as the basic multicellular unit (BMU) and results in the formation of a new osteonal system within the pre-existing bone.^51^ Remodeling occurs first within the host bone and then within the woven bone formed in the peri-implant gap.

Significant damage to the host bone occurs at the time of implantation. This results in microdamage to the bone beyond the site of implantation (1 to 2 mm).^50^ Along with this microdamage, there is an enhanced remodeling within the host bone surrounding the implant, which likely continues for greater than six months.^50^ In addition to host bone remodeling, there is remodeling of the woven bone initially formed within the gap between the implant and the host bone. This results in the formation of a mature lamellar bone.

Formation of the lamellar bone around the implant is the desired end result. Woven bone forms rapidly and consists of loosely packed collagen fibers of varying size in a random spatial alignment. In contrast, the collagen fibers of the lamellar bone are organized in thicker bundles and orientated in the plane of the lamella. This structure makes the lamellar bone mechanically stronger than the woven bone.^52^

Remodeling of the bone in contact with the implant surface continues throughout the lifetime of the implant. This remodeling may allow for increased contact between the implant and bone, with time. Brånemark **et al**., have compared histology with the biomechanical strength of the pull-out and torsion for titanium screws inserted into rat tibia at different time points.^53^ They found that the pull-out strength increased markedly within the first four weeks and this coincided with the bone quantity, which also increased markedly within the first four weeks. Torsion, however, increased only after four weeks. This coincided with the increase in remodeling that occurred within the fourth to sixteenth week period. Furthermore, there was a positive correlation between torsional strength and bone contact with the implant. This implied that remodeling, over time, improved the mechanical interdigitation between the host bone and implant.

Ideally, remodeling will always allow for improved bone bonding between the implant and host bone; however, this is not always the case. Remodeling is significantly influenced by biomechanical stresses within the bone surrounding the implant. Wolf recognized this feature of the bone and stated it in his law: “every change in the function of a bone is followed by certain definite changes in the internal structure and external conformation in accordance with mathematical laws.”^54^ Osteoclastic and osteoblastic activity of the BMU is balanced in healthy bone that is subjected to normal loading resulting from everyday activities (e.g., walking). This balance is lost and the osteoclastic activity predominates when the bone is unloaded for a period of time (e.g., disuse of an extremity due to injury).^51^ This tipping of the scales in favor of osteoclastic activity leads to a loss of bone mass. Similarly, the introduction of an implant changes the stress distribution in the bone and may lead to unbalanced osteoclastic activity of the BMU. This is the mechanism of stress-shielding, which results in bone loss from introduction of an implant that is stiffer than the host bone.^55^

## CONCLUSION

Peri-implant bone healing may be divided into early and late stages. The early stage of healing revolves around the body’s initial response to foreign material: protein adsorption, platelet activation, clot formation, and inflammation. Implant factors that influence the success of healing during this stage are initial implant stability and implant surface design. The implant surface requires a complex topography to allow for bone bonding. Current implant surface designs include sintered porous and plasma-sprayed coatings. HA plasma sprayed porous coated implants offer topographic surface features that enhance osteoconduction and resultant bone bonding. Completion of the early stage of healing results in immature woven bone, bridging the gap between the host bone and the implant surface. This may be accomplished through both contact and distance osteogenesis.

The late stage of bone healing involves remodeling of the host and immature bone, to form a mature lamellar bone. Bone remodeling is a neverending process that occurs throughout the life of the prosthesis. Mechanical stress in the bone surrounding the implant significantly influences this remodeling process.

This review was meant to provide a basic understanding of the bone healing that occurs at an implant’s surface to provide stable anchorage of the implant within the bone. Knowledge of this process is useful to interpret the reasoning behind the orthopedic implant design. Novel implant design features, such as, surface coatings, add cost to implants and it is necessary for the surgeon to evaluate whether such design features warrant the added cost. The clinical outcome data regarding such changes to implant design is often not available for 15 to 20 years after introduction of the implant. Therefore, the surgeon may only be able to evaluate the basic science rationale underlying the change in design of the implant. In the absence of good long-term clinical studies, comprehension of peri-implant healing may help the surgeon with implant selection.
